# iTAR: a web server for identifying target genes of transcription factors using ChIP-seq or ChIP-chip data

**DOI:** 10.1186/s12864-016-2963-0

**Published:** 2016-08-12

**Authors:** Chia-Chun Yang, Erik H. Andrews, Min-Hsuan Chen, Wan-Yu Wang, Jeremy J. W. Chen, Mark Gerstein, Chun-Chi Liu, Chao Cheng

**Affiliations:** 1 Institute of Molecular Biology, National Chung Hsing University, Taichung, 402 Taiwan; 2 Institute of Genomics and Bioinformatics, National Chung Hsing University, Taichung, 402 Taiwan; 3 Institute of Biomedical Sciences, National Chung Hsing University, Taichung, 402 Taiwan; 4 Agricultural Biotechnology Center, National Chung Hsing University, Taichung, 402 Taiwan; 5 Program in Computational Biology and Bioinformatics, Yale University, 260 Whitney Avenue, New Haven, CT 06520 USA; 6 Department of Molecular Biophysics and Biochemistry, Yale University, 260 Whitney Avenue, New Haven, CT 06520 USA; 7 Department of Genetics, Geisel School of Medicine at Dartmouth, Hanover, NH 03755 USA; 8 Geisel School of Medicine at Dartmouth, Institute for Quantitative Biomedical Sciences, Lebanon, NH 03766 USA; 9 Norris Cotton Cancer Center, Geisel School of Medicine at Dartmouth, Lebanon, NH 03766 USA

**Keywords:** Transcription factor, ChIP-seq, ChIP-chip, Gaussian mixture model, Gene ontology analysis

## Abstract

**Background:**

Chromatin immunoprecipitation followed by massively parallel DNA sequencing (ChIP-seq) or microarray hybridization (ChIP-chip) has been widely used to determine the genomic occupation of transcription factors (TFs). We have previously developed a probabilistic method, called TIP (Target Identification from Profiles), to identify TF target genes using ChIP-seq/ChIP-chip data. To achieve high specificity, TIP applies a conservative method to estimate significance of target genes, with the trade-off being a relatively low sensitivity of target gene identification compared to other methods. Additionally, TIP’s output does not render binding-peak locations or intensity, information highly useful for visualization and general experimental biological use, while the variability of ChIP-seq/ChIP-chip file formats has made input into TIP more difficult than desired.

**Description:**

To improve upon these facets, here we present are fined TIP with key extensions. First, it implements a Gaussian mixture model for *p*-value estimation, increasing target gene identification sensitivity and more accurately capturing the shape of TF binding profile distributions. Second, it enables the incorporation of TF binding-peak data by identifying their locations in significant target gene promoter regions and quantifies their strengths. Finally, for full ease of implementation we have incorporated it into a web server (http://syslab3.nchu.edu.tw/iTAR/) that enables flexibility of input file format, can be used across multiple species and genome assembly versions, and is freely available for public use. The web server additionally performs GO enrichment analysis for the identified target genes to reveal the potential function of the corresponding TF.

**Conclusions:**

The iTAR web server provides a user-friendly interface and supports target gene identification in seven species, ranging from yeast to human. To facilitate investigating the quality of ChIP-seq/ChIP-chip data, the web server generates the chart of the characteristic binding profiles and the density plot of normalized regulatory scores. The iTAR web server is a useful tool in identifying TF target genes from ChIP-seq/ChIP-chip data and discovering biological insights.

**Electronic supplementary material:**

The online version of this article (doi:10.1186/s12864-016-2963-0) contains supplementary material, which is available to authorized users.

## Background

Transcription factors (TFs) constitute a family of proteins that play critical roles in regulating gene transcription [1, 2]. Mechanistically, they operate by recognizing and binding specific DNA sequences via DNA-binding domain(s) or by forming complexes with other regulatory co-factors [3]. Owing to the development of technologies such as chromatin immunoprecipitation followed by massive parallel sequencing (ChIP-seq) or DNA hybridization (ChIP-chip), a large number of experiments have in recent years sought to identify genome-wide TF binding sites [4, 5], from which predictions about TF regulatory target genes can be made. Given the ChIP-seq/ChIP-chip data for a TF, several computational methods have been proposed to define these target genes [6–8]. Most of these methods apply a peak-based strategy: first, a peak-calling algorithm [9–11] is employed to generate a list of enriched binding peaks of a TF, with target genes for that TF then defined by analyzing the intensity and location of the binding peaks to nearby genes. As signals from non-significant binding peaks are not considered, these methods are sensitive to the choice of peak-calling method. Indeed, a considerably different set of TF binding peaks and derived target genes are identified when different peak-calling methods or different parameter-settings are used, raising questions of validity and false positivity of this approach.

To address this issue, we have previously developed a probabilistic method, called TIP (Target Identification from Profiles), to identify TF target genes based on ChIP-seq or ChIP-chip data [12]. TIP requires no pre-defined binding peaks; rather, it considers raw experimental TF binding signals from all DNA regions of the genome. TIP works by characterizing a binding profile of a TF around the transcriptional start sites (TSS) of all genes and then uses this profile to weight the binding intensity of a TF in each gene’s promoter region, yielding a continuous-valued binding score of a TF for each gene. TIP then estimates the significance of each gene as a regulatory target by comparing each gene’s binding score to the distribution of all binding scores, using statistical methods that assume distributional normality.

As others and we have shown, TIP identifies TF targets with very high accuracy that can be readily used in downstream biological studies [13]. However, the following issues hinder the application of TIP. First, the assumption that binding scores follow a normal distribution makes the calculation of *p*-values exceedingly conservative, which results in a lower sensitivity of the method for identifying targets. This is because binding scores are generally not normally distributed and instead are either bimodalor positively skewed, reflecting the fact that the binding scores fundamentally encompass two different populations: genuine targets (with higher scores) and background genes (with lower scores). Thus, assuming distributional normality elides over this heterogeneity and loses statistical resolving power. Second, as TIP does not utilize binding peaks, it also does not output them, a shortcoming for downstream analyses as they are helpful for visualization and general experimental biology. Third, while a strength of TIP is that it uses all the track files from ChIP-seq and ChIP-chip experiments, in practice the file format variation of this data (Bed, BedGraph, Wiggle, and BigWig, among others) has made file input into TIP less straightforward than desired.

To address these limitations, here we extend TIP with key modifications. First, were vise the *p*-value calculation using a Gaussian mixture model, thereby more accurately taking into account the shape of the binding score distribution and improving target gene identification sensitivity. Second, we remedy the lack of binding peak output by allowing TIP to optionally incorporate a binding peak file into its analysis and identify peak loci and intensity within the promoter regions of each significant target gene calculated by TIP. Finally, for ease of implementation we have created a web server (http://syslab3.nchu.edu.tw/iTAR/) for track files upload and end-to-end TIP processing. The web server currently accepts Wig, BigWig, and BedGraph compressed (.gz or.rar) formats from TF ChIP-seq/ChIP-chip experiments as the input, which contains the binding signals of a TF at each genomic position (e.g., raw/normalized read coverage or fold-change), and enables analysis for seven organisms (human, mouse, fly, worm, chicken, zebrafish and yeast) with multiple genome assembly support for human and mouse. With a user-friendly interface and extensible backend for future genome version support, the server is widely usable for TF target gene identification and ChIP-seq analysis going forward.

As a walkthrough of its capabilities we provide an example of its use for STAT3 ChIP-seq data. A validation of its output is conducted using the NFE2 ChIP-seq and gene expression dataset.

## Construction and content

### Server construction, supported organisms and genome assemblies

The iTAR web server runs on Linux and is implemented in JSP and Java. ChIP-seq data analysis is provided for seven different organisms: human, mouse, fly, worm, chicken, zebrafish and yeast, with multiple genome assemblies available for human (UCSC hg 18 and hg19) and mouse (UCSC mm8, mm9, and mm10). In addition, support for genome assemblies is extensible to enable further genome assembly availability going forward.

Annotation files for RefSeq genes are downloaded from the UCSC Genome Browser [14]. These files provide the chromosome, strand, transcriptional start site, transcription terminal sites, structures and other genomic information for all RefSeq genes for an organism, based on the corresponding genome assembly.

The server is freely available to the public and does not require login. Analysis of a ChIP-seq dataset for target gene identification using the server is straightforward and requires only three steps: (i) uploading input files, (ii) choosing parameters, and (iii) receiving the output files.

### Input files

The iTAR web server takes as input a ChIP-seq/ChIP-chip signal track file and optionally a binding peak file for a TF. The signal track file contains the binding signal of a TF at each nucleotide position of the genome as measured by sequencing in ChIP-seq or hybridization in ChIP-chip data. The server supports three signal track formats: BedGraph, Wiggle, and BigWig. We note that each format may have multiple variants. For example, Wiggle files have two main formatting options--fixedStep and variableStep, designed for data with regular and irregular intervals between data points, respectively. The server contains a module to process different formats of signal track files to ensure the best usability.

The optional peak file is in bed format, containing the chromosome, start position, end position and other information of TF binding peaks. Users can run a peak calling program, e.g. MACS or PeakSeq [9, 11], using the uploaded ChIP-seq data as input to generate this peak file.

To improve the uploading time, the signal track files must be compressed into either.rar or.gz format before uploading. Alternatively, user can support download links for track file and peak file, and then iTAR will download the input files automatically.

### Parameter settings

**Fig. 1:**
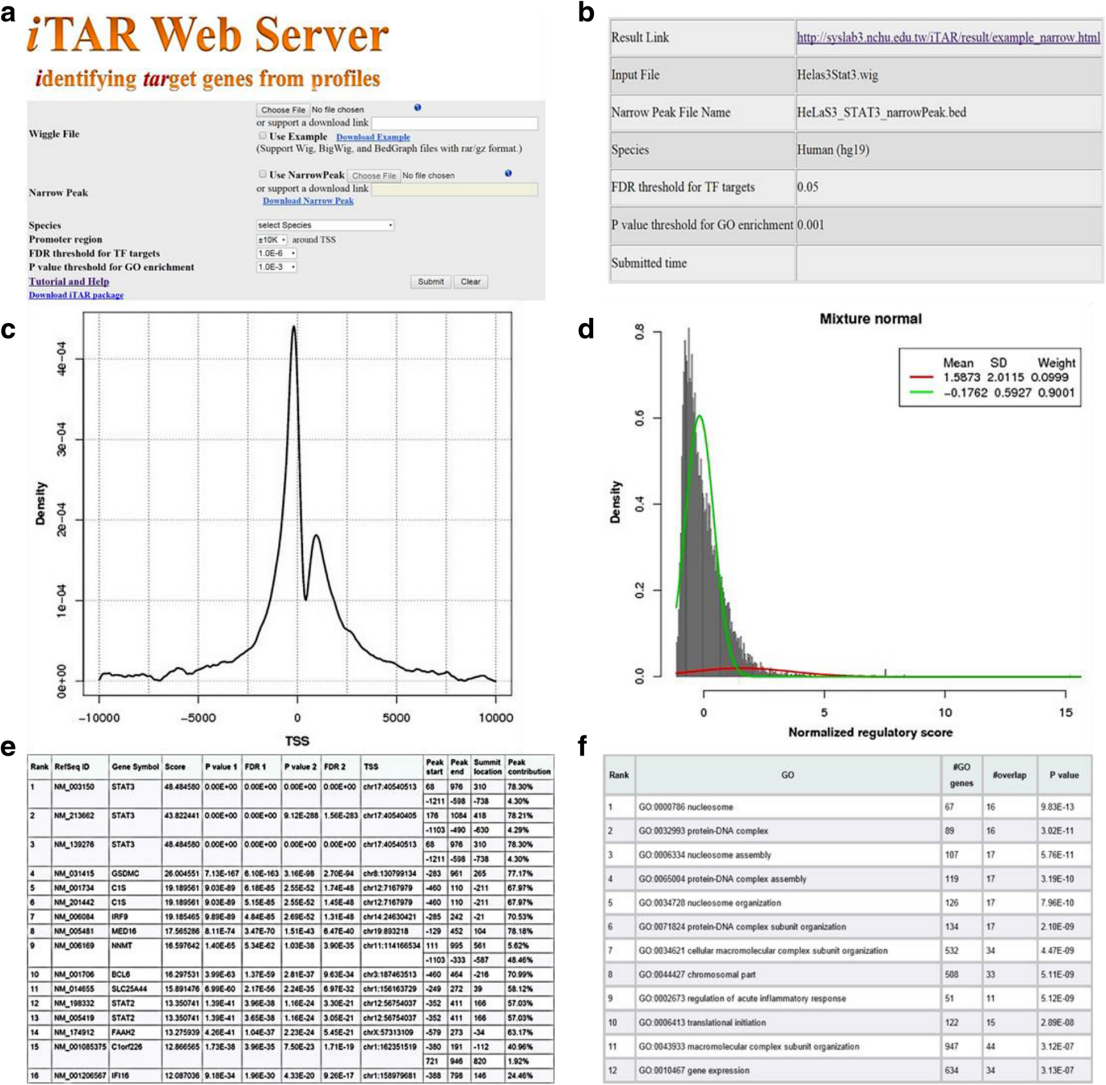
An overview of the iTAR web server. **a** The portal page of the iTAR web server: wig, bigwig, and bedgraph files in rar or gz format can be used for upload. **b** The waiting page: after a wiggle file is uploaded, a waiting page will be shown to the user, updating job status every 5 s. For this example, we used the ENCODE STAT3 ChIP-seq data in HeLa-S3 cells. **c** The characteristic binding profile of an example TF. It shows the aggregation plot of TF binding in a 20 kb DNA region centered at the transcriptional start site (TSS). This plot can be used to make a rough evaluation of the data quality. In general, we expect to see a peak near by the TSS. **d** The density plot of normalized regulatory scores (Z score). For each gene, the iTAR server will calculate a regulatory score, measuring the binding strength of a TF to the gene. The regulatory scores for all genes will then be normalized, and p-values will be calculated based on a single normal distribution model or a mixture normal distribution model. **e** A list of significant target genes and the associated TF binding site: significant RefSeq genes, gene symbols and *P*-values will be shown. *P*-value 1 is estimated based on the mixture normal distribution model, and *P*-value 2 is estimated based on the single normal distribution model. The summit location is the summit of the binding peak from TSS. **f** GO enrichment analysis with the target genes

After the input file(s) have been uploaded, users need to select the organism and the genome assembly version from the drop-down list (see Fig. 1a). The selected organism/assembly must match with the uploaded signal track file and an optional peak file to ensure accurate results in subsequent analysis. In addition, users need to specify three other parameters: the length of considering binding signals around TSS, the FDR (false discovery rate) threshold for TF targets and the *P*-value threshold for GO enrichment analysis. The length of promoter region will be used to select the regions of the putative promoters, the FDR threshold will be used to select a subset of target genes of the TF for subsequent GO enrichment analysis, and the *P*-value threshold will be used to select significantly enriched GO categories for displaying in the output webpage. After all parameters have been specified, users can click the “Submit” button to initiate online data analysis. A waiting page will be displayed and updated every 5 s to report the progress of the data analysis (Fig. 1b). Depending on the organism and the signal track file format, analysis may take 10–25 min to finish.

### Output files

After data analysis has completed, the results will be displayed in an output webpage. The main output consists of five panels that include (i) a table summarizing the parameter and input settings, (ii) a characteristic binding profile of the TF, (iii) the distribution of normalized regulatory scores for all genes, (iv) a list of significant genes and (optionally) their associated TF peak binding sites, and (v) a list of significantly enriched GO terms (see Fig. 1 for an example). Significant genes in (iv) are sorted by decreasing order of their regulatory scores. For each gene, the *p*-value and the multiple-testing corrected FDR value are calculated, using both single normal and mixture normal models. To enable parameter setting adjustment and iterative analyses based on the output results, a “Rerun the program” option is included that accepts parameter setting modifications (to change statistical stringency requirements for GO analysis, for example) without the need for re-uploading input files.

### TIP extensions

As mentioned above and previously described, TIP builds a characteristic, averaged binding profile for a TF around the TSS of all genes and then uses this profile to weight the sites associated with a given gene, providing a continuous-valued regulatory score of a TF for each gene. It then normalizes the regulatory scores into z-scores and estimates their *p*-values by referring to a standard normal distribution [12]. However, using a standard normal distribution to estimate the significance of regulatory scores is highly conservative, giving rise to a confident but small target gene set. This is because the distribution of the regulatory scores is typically not normal but positively skewed (Fig. 1d) or bimodal (Additional file 1: Figure S1), reflecting the fact that binding scores encompass two distinct groups: background, non-target genes with low regulatory scores, and genuine target genes with higher regulatory scores. By not taking this non-normal distributional shape into account, statistical resolution is lost and *p*-value estimates are conservative.

Motivated by these observations, here we refine TIP’s *p*-value calculation by applying a two-component Gaussian mixture model to estimate the significance of normalized regulatory scores. Our approach is as follows: suppose that each regulatory score *y*_1_, …, *y*_*n*_ is instead derived from a two-component (i.e., non-target and genuine target genes, respectively) Gaussian mixture distribution. The log-likelihood for data consisting of *n* observations *y* = (*y*_1_,…,*y*_*n*_), assuming a normal mixture model with two components, is then given by

1 $$ \ell \left(\varTheta \Big|\boldsymbol{y}\right)={\displaystyle \sum_{i=1}^n} \log \left({\displaystyle \sum_{k=1}^2}{w}_kN\left({\mathrm{y}}_i\Big|{\mu}_k,{\sigma}_k\right)\right) $$

where *Θ* = (**w**, *Ψ*) represents all unknown parameters and *N*(⋅|*μ*_*k*_, *σ*_*k*_) denotes a Gaussian density function with mean *μ* and standard deviation *σ*. Here the vector *w* = (*w*_1_, *w*_2_) consists of the mixing proportions and subjects to ${\displaystyle \sum_{k=1}^2}{w}_k=1$, *Ψ* = (*μ*_1_, *μ*_2_, *σ*_1_, *σ*_2_). The maximum likelihood estimation of *Θ* can then be solved by

2 $$ \widehat{\varTheta}= \arg \underset{\varTheta }{ \max}\ell \left(\varTheta \Big|\boldsymbol{y}\right) $$

To choose the best mixture model with optimal parameters of the two Gaussian distributions, we use an expectation-maximization (EM) algorithm [15] (See Fig. 1d). EM is an iterative method for finding maximum likelihood estimation of parameters by using the following iterative algorithm,

3 $$ {\widehat{w}}_k=\frac{{\displaystyle {\sum}_{i=1}^n}{\widehat{z}}_{ik}}{n},{\widehat{\mu}}_k=\frac{{\displaystyle {\sum}_{i=1}^n}{\widehat{z}}_{ik}{\mathrm{y}}_i}{{\displaystyle {\sum}_{i=1}^n}{\widehat{z}}_{ik}},\kern0.5em {\widehat{\sigma}}_k^2=\frac{{\displaystyle {\sum}_{i=1}^n}{\widehat{z}}_{ik}{\left({\mathrm{y}}_i-{\widehat{\mu}}_k\right)}^2}{{\displaystyle {\sum}_{i=1}^n}{\widehat{z}}_{ik}} $$

with the posterior probabilities

4 $$ {\widehat{z}}_{ik}=\frac{{\widehat{w}}_kN\left({\mathrm{y}}_i\Big|{\widehat{\mu}}_k,{\widehat{\sigma}}_k\right)}{{\displaystyle {\sum}_{h=1}^2}{\widehat{w}}_hN\left({\mathrm{y}}_i\Big|{\widehat{\mu}}_h,{\widehat{\sigma}}_h\right)} $$

Once the optimal parameters are determined, we estimate *p*-values by comparing each z-score to the left Gaussian distribution (with the smaller mean value).

### Gene ontology enrichment analysis

Given the target genes, we performed enrichment analysis with GO terms using the Fisher exact test based on a hypergeometric distribution [16]. To exclude non-informative general GO terms, we restricted our analysis to those with gene numbers < 1000. The web server shows significant GO terms ranked by *p*-value and provides the predicted target genes from them.

## Utility and discussion

### STAT3 ChIP-seq data

The ENCODE project contains STAT3 ChIP-seq data for experiments run in HeLa-S3 cells [5]. By checking the “Use Example” box and then clicking “Submit” in the interface page of the iTAR server (Fig. 1a), ChIP-seq data for STAT3 is processed and the results displayed in an output page. The output page contains five panels (Fig. 1b-f). The first panel shows a table that summarizes the data analysis including organism, version of genome assembly, parameter settings and submission time (Fig. 1b). The second panel shows the characteristic binding profile for STAT3, also referred to as an aggregation plot, which shows the average binding signals of STAT3 across all RefSeq genes in the +/− 10 kb DNA regions centered at the TSS of each gene (Fig. 1c). As shown, the profile displays a sharp peak around the TSS, suggesting that STAT3 shows a strong binding preference to the TSS proximal regions. While in general Fig. 1c’s purpose is to enable a visual check of binding profile characteristics, the location hints at underlying biology. Although most TFs show enriched binding signals around the TSS of genes, their characteristic binding profiles vary. This suggests that (i) different TFs tend to bind at different locations relative to TSSs, which could affect their transcriptional regulation of their target genes – signals closer to TSS may contribute more to their target gene regulation [8, 17]; (ii) the binding signals of different TFs at the same location might influence nearby gene transcription differently -- some TFs may exert their influence over long distances, while others exert only regional effects. Overall, Fig. 1c thus enables the study of overall trends of a TF’s regulation across the genome.

The third panel shows the distribution of normalized regulatory scores of STAT3 on all human RefSeq genes (Fig. 1d). The distribution shows a long tail to the right side, which can be decomposed into two separate distributions by using a two-component Gaussian mixture model (the red and the green curves). The main output of the iTAR web server is a ranked gene list as shown in the fourth panel (Fig. 1e), with genes sorted in decreasing order of their regulatory scores. *P*-values of all genes are calculated and then adjusted using the Benjamini–Hochberg multiple testing correction method (i.e. FDR) based on a single normal distribution as well as a mixture normal distribution. When the single normal distribution is used, a total of 241 STAT3 RefSeq target genes are identified at the 0.05 significance level (FDR < 0.05). In contrast, the method based on mixture normal distribution identifies 614 RefSeq target genes at the same significance level, highlighting its increased sensitivity of target gene prediction. By integrating Fig. 1d with 1e, users can analyze the binding profile distribution and decide which distribution model and *p*-value calculation works best for their data and application.

The fifth panel is a table containing results from GO enrichment analysis (Fig. 1f) of STAT3’s predicted target genes. Previous work has reported that unphosphorylated STAT3 (U-STAT3) influences gene transcription in response to cytokines [18]. In *Drosophila*, U-STAT92E is associated with HP1 and maintains heterochromatin stability. In addition, the U-STAT3-DNA interaction structure is important for chromatin organization [19]. Taken together, U-STAT3 is considered to function as a transcriptional activator of immune cells and a chromatin organizer. A total of 185 GO terms are significantly enriched in STAT3 target genes, in which the first, third, and fifth GO terms are associated with the nucleosome (GO:0000786, GO:0006334 and GO:0034728) and the ninth GO term is associated with the immune response (i.e. GO:0002673), consistent with STAT3’s known functionality. GO:0000786 nucleosome has 67 genes, among which 16 genes are STAT3 target genes (P = 1E-12) (Fig. 1f). To demonstrate the STAT3 binding signal in these 16 genes, Additional file 1: Figure S2 shows the STAT3 binding profiles in the promoter regions.

**Fig. 2:**
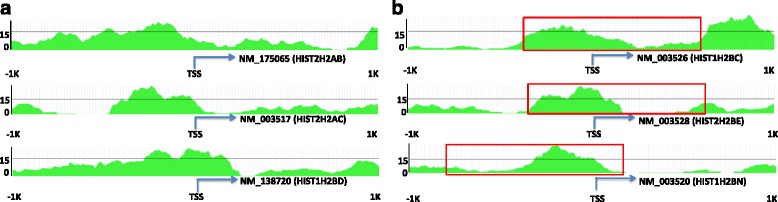
The STAT3 binding profiles in the promoter regions of six histone proteins. The binding profiles are generated by using STAT3 ChIP-seq data in HeLa-S3 cells from the ENCODE project. We selected six histone proteins from STAT3 target genes using the TIP algorithm. The red rectangles indicate peaks from PeakSeq method. **a** There is no significant peak using PeakSeq method but TIP identifies the genes as target genes. **b** There are significant peaks using PeakSeq method in the promoter regions and TIP also identifies the genes as the target genes

Among these 16 genes, some TF target genes have one or more strong binding peaks in their promoter regions (Fig. 2b), which can be identified by both TIP and peak-based methods. On the other hand, some TF targets are identified by TIP but not by peak-based methods (Fig. 2a). As shown, these target genes are also associated with one or more binding signals, but they are relatively weak and not identified as significant peaks by peak-calling methods. Thereby, TIP shows a higher sensitivity in detecting TF target genes with weak but considerable binding signals.

### The comparison of single normal distribution and mixture normal distribution

**Fig. 3:**
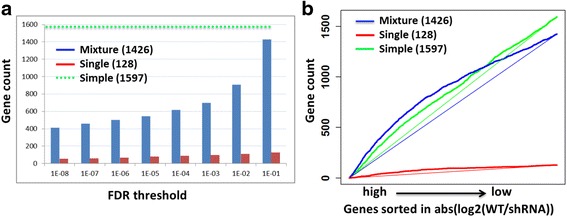
Comparison analysis of target genes identified by single normal distribution and mixture normal distribution. We utilized the NFE2 ChIP-seq data and gene expression data treated by NFE2 shRNA in K562 cells. **a** We compared the identified NFE2 target genes using three methods as follows: simple method, TIP with single normal distribution and TIP with mixture normal distribution. In the simple method, we identified target genes using a conventional peak-based method. The simple method identified 1597 target genes. We used various FDR thresholds (x axis) of TIP algorithm to select significant NFE2 target genes. The y axis represents the number of target genes. **b** We calculated the expression changes (absolute value of log ratios) of all genes for the cells treated with shRNA as compared to the untreated cells, and then we sorted the genes in decreasing order of absolute value of their log ratios. The genes on the left have greater absolute value of log ratios (WT vs. shRNA) and are therefore more responsive to NFE2 regulation. Given a threshold of absolute log ratio (x axis), the y axis shows the number of target genes satisfied the absolute log ratio threshold for each method

To demonstrate the advantage of our extended TIP method with mixture normal distribution, we utilized the NFE2 ChIP-seq data and gene expression data treated by NFE2 shRNA in K562 cells from the ENCODE project [5]. We compared the identified NFE2 target genes using three methods as follows: simple method, TIP with single normal distribution and TIP with mixture normal distribution. In the simple method, we identified target genes using a conventional peak-based method: select target genes as those containing one or more binding peaks in their proximal (+/− 1000 bp of the TSS) promoter regions. The simple method identified 1597 target genes. A total of 128 target genes are identified for NFE2 by TIP at a FDR <0.1 significance threshold when employing a single normal distribution for significance testing. In contrast, the mixture normal based analysis identifies 1426 target genes at the same significance level. Figure 3a shows the number of significant target genes with various FDR thresholds of TIP algorithm. The TIP with single normal distribution is a stringent method and the numbers of significant target genes are stable but much less than the other methods, whereas the TIP with mixture normal distribution provides reasonable numbers of target genes.

For target validation, we calculated the expression changes (absolute value of log ratios) of all genes for the cells treated with shRNA as compared to the untreated cells, and then we sorted the genes in decreasing order of absolute value of their log ratios. As shown in Fig. 3b, genes on the left have greater absolute value of log ratios (WT vs. shRNA) and are therefore more responsive to NFE2 regulation. Given a threshold of absolute log ratio (x axis), the y axis shows the number of target genes satisfied the absolute log ratio threshold for each methods. The area between the gene number curve and the straight line indicates accuracy of the target gene prediction. The area of the TIP with mixture normal distribution (the area between blue curve and blue straight line) has larger area than the other two methods. As shown, target genes identified by TIP are significantly more responsive to regulate by NFE2 than those identified by the conventional peak-calling method. Estimating *p*-value of genes based on mixture normal distributions increases the sensitivity of TIP, resulting in a larger target gene set than that based on the single normal distribution.

### The comparison of read-coverage and fold-change signals of input data

To compare read-coverage and fold-change signals of input data, we downloaded the K562 Nfe2 ChIP-seq data and ChIP-seq control from the ENCODE project and then use bowtie to re-align the reads (parameters: −m 1 -n 2 -S -a --best --strata) [20]. Using bamCoverage and bamCompare tools of deepTools software (version 1.5.9.1) [21], we obtained signal files of the read coverage and fold change, respectively. 997 and 502 target genes were identified for read-coverage and fold-change signal files, respectively, by iTAR with mixture normal distribution (FDR <0.1). Additional file 1: Figure S3 shows the cumulate distribution of target genes identified by iTAR using read-coverage and fold-change signals. As shown, the target genes identified by iTAR using read-coverage signals is more responsive to NFE2 regulation.

## Conclusions

With the rapidly increasing accumulations of ChIP-seq data, we refined the TIP algorithm to construct the web server, which offers a simple and user-friendly interface for experimental biologists. We believe that the iTAR web server will accelerate the research of gene regulation and help the experimental biologists to discover important biological mechanism. In addition, we provide a stand-alone JAVA package that users can choose to download and analyze their ChIP-seq data in their computers.

iTAR supports GO enrichment analysis for target gene list. In addition, users can download the predicted target gene list from iTAR, and submit to other interesting database.

## Availability and requirements

Project name: iTAR

Project home page: http://syslab3.nchu.edu.tw/iTAR/

Operating system: Linux

Programming language: R and Java

Other requirements: R version > = 2.10.0, R package: segmented, nor1mix (version > =1.2) and mixtools.

Physical memory > = 12GB

## Abbreviations

ChIP-seq, chromatin immunoprecipitation followed by massively parallel DNA sequencing; FDR, false discovery rate; iTAR, a web server for identifying target genes of transcription factors using ChIP-seq or ChIP-chip data; TF, transcription factor; TIP, target identification from profiles; TSS, transcriptional start sites

## Additional file

Additional file 1:
**Figure S1.** The density plot of normalized regulatory scores (Z score) using the ENCODE Stat1 ChIP-seq data in GM12878 cell line. **Figure S2.** The STAT3 binding profiles in the promoter regions of 16 histone proteins. **Figure S3.** Comparison of target genes identified by iTAR using read-coverage and fold-change signals. (DOCX 544 kb)
