# Supplementary material for: iTAR: a web server for identifying target genes of transcription factors using ChIP-seq or ChIP-chip data
Source: BMC Genomics. 2016 Aug 12;17:632. doi: 10.1186/s12864-016-2963-0 (PMC4983039; doi:10.1186/s12864-016-2963-0)
Supplement: Additional file 1: — Figure S1. The density plot of normalized regulatory scores (Z score) using the ENCODE Stat1 ChIP-seq data in GM12878 cell line. Figure S2. The STAT3 binding profiles in the promoter regions of 16 histone proteins. Figure S3. Comparison of target genes identified by iTAR using read-coverage and fold-change signals. (DOCX 544 kb) [file 12864_2016_2963_MOESM1_ESM.docx]

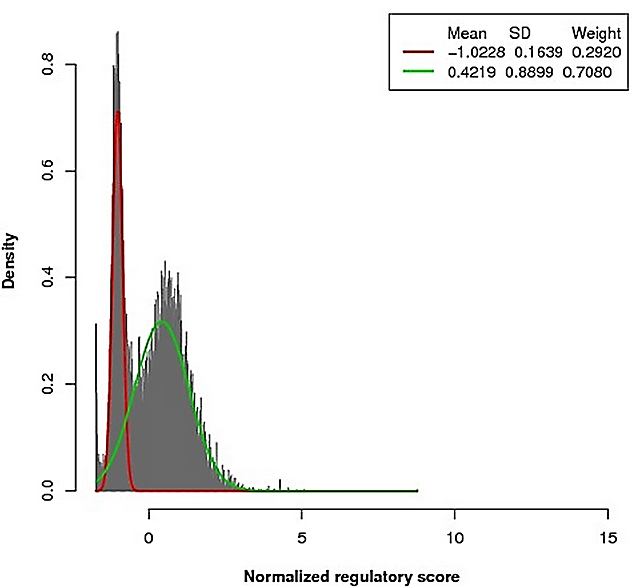


**Additional file 1: Figure S1.** The density plot of normalized regulatory scores (Z score) using the ENCODE Stat1 ChIP-seq data in GM12878 cell line. For each gene, the TIP server will calculate a regulatory score, measuring the binding strength of a TF to the gene. The regulatory scores for all genes will then be normalized, and P-values will be calculated based on a single normal distribution model or a mixture normal distribution model.

**Additional file 1: Figure S2.** The STAT3 binding profiles in the promoter regions of 16 histone proteins. The binding profiles are generated by using STAT3 ChIP-seq data in HeLa-S3 cells from the ENCODE project. We selected 16 histone proteins from STAT3 target genes using TIP algorithm. The red rectangles indicate peaks from PeakSeq method.

**Additional file 1: Figure S3.** Comparison of target genes identified by iTAR using read-coverage and fold-change signals. 997 and 502 K562 NFE2 target genes are identified for read-coverage and fold-change signals by iTAR with mixture normal distribution (FDR <0.1), respectively. Cumulative distribution of genes in the gene list is sorted by expression changes. The genes on the left have greater absolute value of log ratios (WT vs. shRNA) and are therefore more responsive to NFE2 regulation.
